# The effects of play-based interventions on social and play skills in children with autism spectrum disorder: a meta-analysis

**DOI:** 10.1007/s11845-026-04477-x

**Published:** 2026-06-13

**Authors:** Yeter Çuvadar Baş, Semra Karaca, Esra Demi̇rci̇ Ecevi̇t

**Affiliations:** 1 Department of Psychiatric Nursing, Marmara University, Istanbul, Turkey; 2 Department of Pediatric Health and Diseases Nursing, Medipol University, Istanbul, Turkey

**Keywords:** Children, Game therapy, Autism, Skill

## Abstract

**Background:**

Autism Spectrum Disorder (ASD) is characterized by deficits in social communication and interaction, along with restricted and repetitive behaviors. Play-based interventions are widely used to enhance social and play skills in children with ASD; however, their overall effectiveness remains unclear.

**Aims:**

This meta-analysis aimed to evaluate the effects of play-based interventions on social and play skills in children with ASD.

**Methods:**

A systematic search was conducted in PubMed, Web of Science, EBSCOhost, YOK Thesis, and Google Scholar between June and October 2024. Study quality was assessed using Joanna Briggs Institute appraisal tools appropriate to each design. Seven studies met the inclusion criteria. Data were analyzed using Comprehensive Meta-Analysis (CMA) Version 2. Standardized mean differences (SMD) with 95% confidence intervals (CI) were calculated using meta-analytic techniques, supported by narrative synthesis.

**Results:**

Play-based interventions were significantly associated with improvements in social skills (SMD = -1.774, 95% CI: -3.088 to -0.461; *p* = 0.008; I² = 93.730) and play skills (SMD = 1.518, 95% CI: 0.237 to 2.798; *p* = 0.020; I² = 94.768). Subgroup analyses showed that Child-Centered Play Therapy (CCPT), Image-Sand Play Therapy (ISPT), and Sensory Integration-Based Play Therapy (SIBPT) demonstrated comparatively larger effect sizes. Interventions delivered by trained therapy professionals yielded greater effectiveness. High heterogeneity was observed across studies.

**Conclusions:**

Play-based interventions appear effective in improving social and play skills in children with ASD; however, findings should be interpreted cautiously due to substantial heterogeneity. Further high-quality studies are warranted.

## Introduction

Autism Spectrum Disorder (ASD), a neurodevelopmental disorder, is characterized by significant limitations in critical developmental areas, including communication, social interaction, and play [1, 2]. Childhood is one of the pivotal periods during which individuals develop social skills and play abilities. In children with ASD, deficits in social communication and limitations in play skills hinder peer interactions and may lead to social isolation [3]. Such challenges not only negatively affect emotional well-being but may also result in lower self-confidence and delays in the development of social competencies. The social exclusion of individuals with ASD contributes to inequities in access to opportunities (from education to employment) and leads to insufficiencies in social integration. Consequently, decreased quality of life and limited societal participation impose both psychological and financial burdens on families while also fueling broader social fragmentation and stigma [2, 3].

Play-based interventions constitute a broad category of therapeutic approaches that utilize play as a primary medium to support children’s social, emotional, and developmental functioning. Within this framework, play may be employed through different theoretical orientations, ranging from child-centered and nondirective play therapy models to more structured developmental or behavioral approaches that integrate play-based interaction strategies. In children with ASD, play-based interventions are frequently used due to their potential benefits in promoting social interaction, communication skills, and emotional regulation [4–6]. These interventions may support children in modeling real-life situations and adopting various social roles through symbolic or interactive play, thereby facilitating opportunities for spontaneous social engagement and problem-solving during play activities [7, 8].

To date, numerous studies have reported that play-based therapeutic approaches are associated with improvements in the social and play skills of children with ASD. For example, group-based play interventions have been shown to enhance abilities such as empathy, social awareness, and social adaptability [1, 2, 7]. However, the existing literature in this field largely consists of individual trials or small-scale studies, and the diversity of intervention models and outcome measures poses challenges for drawing overarching conclusions. Although various play-based approaches have been employed across studies, the number of systematic reviews and meta-analyses that comprehensively synthesize their effects remains limited [8–10].

Importantly, previous reviews have generally focused on specific intervention modalities or narrowly defined therapeutic approaches, rather than integrating the broad spectrum of play-based interventions used for children with ASD [8–10]. In addition, earlier meta-analyses have provided limited examination of potential moderators such as practitioner characteristics and intervention subtypes, making it difficult to determine whether certain play-based approaches may be associated with stronger outcomes than others. Therefore, there remains a need for a more comprehensive synthesis that comparatively evaluates different play-based intervention models within a unified analytical framework. This meta-analysis aims to quantitatively synthesize the effects of play-based interventions on social and play skills in children with autism spectrum disorder, and to examine potential moderators such as intervention type and practitioner characteristics in order to provide a more nuanced and evidence-based understanding of their effectiveness. By integrating findings from diverse intervention models, the study provides a structured and comparative evaluation of play-based therapeutic approaches while addressing gaps related to intervention heterogeneity and practitioner characteristics. In addition, the findings are expected to offer cautious and evidence-informed guidance for practitioners regarding which play-based approaches may be associated with stronger outcomes.

## Methods

This meta-analysis was developed in accordance with the PRISMA checklist [11]. This review was prospectively registered in the PROSPERO database (CRD420251021046). To minimize the bias risk, the processes of literature search, study selection, and data extraction were carried out by two separate researchers independently. Data extraction, quality assessment, and study eligibility decisions were independently performed by two reviewers, and any discrepancies were resolved through discussion and consensus. The quality of the studies included was carefully assessed and verified by the researchers.

### Inclusion and exclusion criteria

This Systematic Literature Review (SLR) and Meta-Analysis incorporates data from randomized controlled trials (RCTs) that examined the effects of play-based interventions on children with ASD. In this review, play-based interventions were defined as therapeutic approaches that utilize play as a primary medium for developmental, behavioral, or psychosocial support. These interventions may include both child-centered play therapy models and structured naturalistic developmental behavioral interventions that incorporate play-based interaction strategies. Although the included intervention models differed in their theoretical orientation and implementation procedures, they were grouped under the broader category of “play-based interventions” because all interventions shared the common characteristic of using play as the primary therapeutic and interactional medium to enhance children’s social communication, engagement, and play-related abilities. Furthermore, potential differences between intervention types were explored through subgroup and moderator analyses in order to better understand variations in intervention effectiveness. The included studies focused on outcome measures related to social and play skills. All studies met the predefined PICOS (Population, Intervention, Comparators, Outcomes, Study design) eligibility criteria.

### PICOS criteria

#### Population (P)

Children diagnosed with ASD who exhibit deficiencies in social and play skills. Exclusion: Studies that did not report outcomes.

#### Intervention (I)

Play-based therapeutic interventions, including both individual and group formats, employing structured or unstructured play techniques (e.g., CCPT, IBSPT, SIBPT, JASPER-EMT). These intervention approaches were collectively evaluated within a unified play-based framework due to their shared emphasis on therapeutic play interaction and developmental engagement. However, recognizing the conceptual and procedural differences among intervention models, moderator analyses were conducted to examine whether intervention type contributed to variations in outcomes.

#### Comparison (C)

Groups not receiving play-based intervention or no intervention (control groups).

#### Outcomes (O)

Studies have to report at least one of the following outcomes along with a measure of variability (e.g., SD, 95% CI): changes in social skill levels, improvements in play skills. Across the included studies, these outcomes were assessed using a variety of methods, including standardized assessment scales, observational evaluation tools, and caregiver- or practitioner-reported measures related to children’s social interaction and play behaviors. Given the variability in measurement instruments across studies, standardized mean differences (SMDs) were calculated to ensure comparability of effect sizes within the meta-analytic framework.

#### Study design (S)

RCTs involving the specified intervention or comparator, published in either Turkish or English.

#### Exclusion

Letters to the editor, quasi-experimental studies, case reports, case presentations, qualitative studies, systematic or descriptive studies, narrative reviews, and studies published before 2014 were excluded from this review.

### Search strategy

Between June and October 2024, searches were conducted in Google Scholar, PubMed, EBSCOhost, and Web of Science databases for the last 10 years using the following MeSH-compatible keywords: [(“Autism” OR “ASD” OR “Autism Spectrum Disorder”) AND (“Child”) AND (“Play Therapy” OR “Play-Based Intervention”) AND (“Social Skills” OR “Communication Skills” OR “Relational Skills” OR “Behavioral Skills”)]. Both free-text keywords and MeSH-compatible terminology were used to maximize the sensitivity of the search strategy. Searches were limited to studies published in English or Turkish between 2014 and 2024. Relevant studies were imported into Mendeley for further screening and analysis.

### Selection of studies

**Fig. 1:**
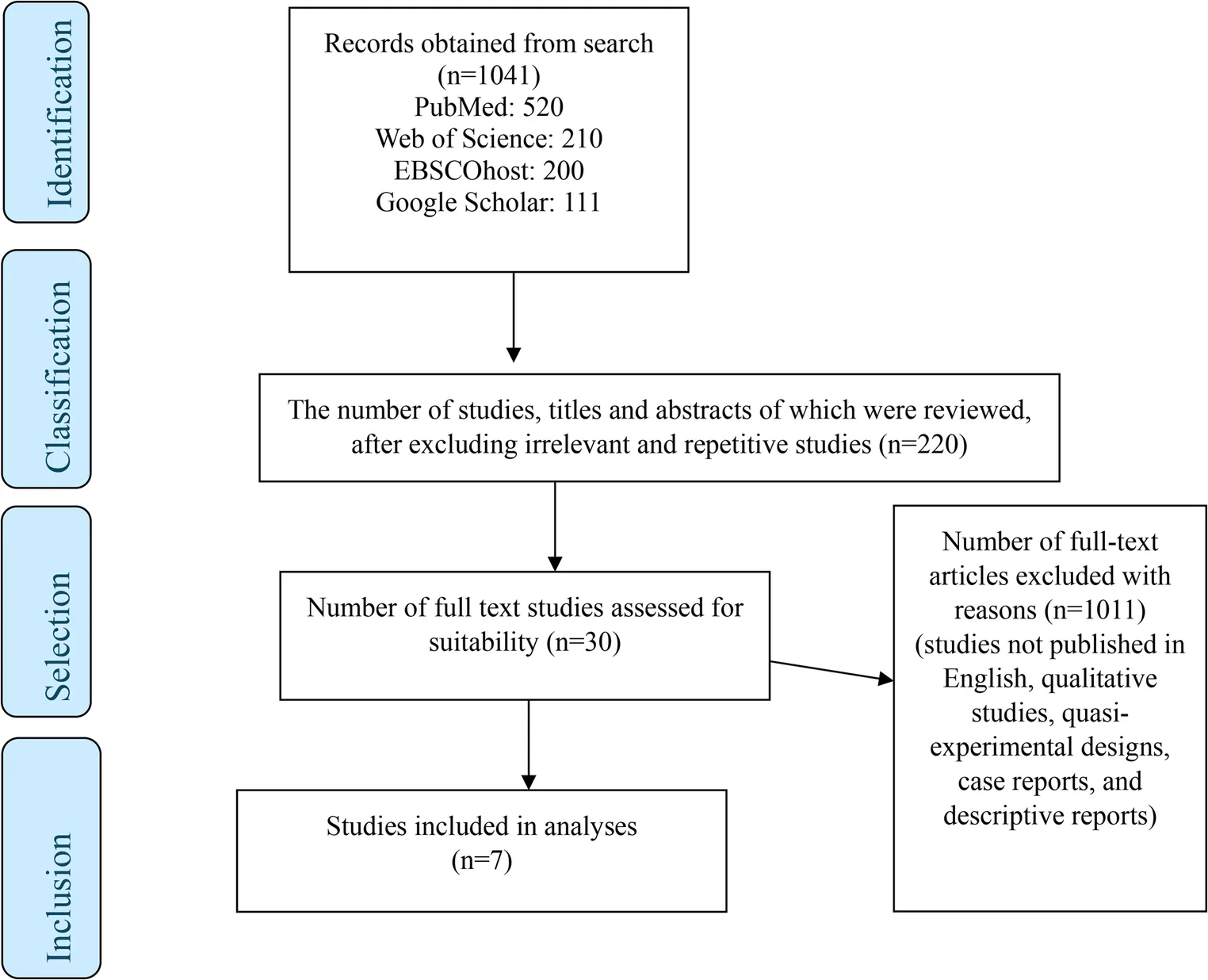
Selection of studies by using PRISMA

The initial search yielded 1,041 records. After excluding irrelevant studies and duplicates, 220 studies’ titles and abstracts were examined in detail. Title/abstract screening and full-text eligibility assessments were independently conducted by two reviewers. Full-texts of 20 studies were retrieved and subjected to a thorough evaluation. Moreover, 30 full-text articles were examined considering the inclusion and exclusion criteria. Ultimately, 7 studies examining the effects of play therapy on the social and play skills of children with ASD were included in the final analysis. Figure 1 illustrates a detailed flowchart of the study selection process.

### Data extraction

To facilitate data collection and organization, the researchers developed a custom data extraction tool. This tool enabled the systematic compilation of data from the studies included in this review and ensured their integration into the meta-analytic process. Information extracted included characteristics of intervention and control groups, mean post-test scores, standard deviations, authorship, year of publication, country of origin, sample sizes, patient groups, assessment tools, key findings, and quality scores. All extracted data were independently verified by two reviewers to reduce transcription errors and reviewer bias. These data were systematically recorded using the tool (Table 1).

**Table 1:** Characteristics and results of the included studies

Author/Year	Country/Continent	Sample Size Characteristics	Assessment Tool	Main Findings	Intervention	Quality Score
Chang et al., 2016	USA	Intervention: 38 Control: 28	Structured Play Assessment (SPA)	Teachers can effectively implement evidence-based interventions, which in turn lead to significant improvements in the core deficits of children with ASD.	JASPER training	Yes:12/13 No:0/13 Uncertain:1/13 Not applicable:0/13
Chang et al., 2017	USA	Intervention:29 Control:29	JASPER Play Assessment Scale	Minimally verbal children with ASD can strengthen their play skills through a targeted intervention program, which also supports symbolic play development and progress in expressive language skills.	CCPT	Yes:11/13 No:1/13 Uncertain:1/13 Not applicable:0/13
Guo & Li, 2021	China	Intervention:45 Control:45	Aberrant Behavior Checklist (ABC),	Intervention group had lower post-intervention ABC scores compared to the control (*P* < 0.05).	IBSPT	Yes:9/13 No:3/13 Uncertain:1/13 Not applicable:0/13
Rabiei et al., 2024	Iran	Intervention:15 Control:15	Gilliam Autism Rating Scale-2 (GARS-2)	Findings indicate that SIBPT offers goal-directed play activities tailored to each child’s unique sensory needs, enhancing adaptive responses to stimuli and contributing to functional improvements in children with ASD.	SIBPT	Yes:9/13 No:4/13 Uncertain:0/13 Not applicable:0/13
Ritzi et al., 2017	Australia	Intervention:12 Control:12	Child Behavior Checklist–Parent Report	Both parents and teachers reported significant reductions in externalizing problem behaviors in children who received intensive CCPT.	CCPT	Yes:9/13 No:4/13 Uncertain:0/13 Not applicable:0/13
Roberts et al., 2022	USA	Intervention:58 Control:62	Socially Communicative Acts	The caregiver-focused intervention, which aimed to teach strategies for supporting social communication twice weekly, did not result in significant outcomes for the children.	JASP-EMT (Joint Attention, Symbolic Play, Engagement & Regulation + Enhanced Milieu Teaching):	Yes:10/13 No:3/13 Uncertain:0/13 Not applicable:0/13
Schottelkorb et al., 2019	USA	Intervention:12 Control:11	Social Responsiveness Scale–2nd Edition (SRS-2)	Intensive CCPT was determined to be effective in decreasing both core autism symptoms and behavioral problems in children diagnosed with ASD.	CCPT	Yes:10/13 No:3/13 Uncertain:0/13 Not applicable:0/13

### Research ethics

As this study is a meta-analysis on published literature, no ethical approval was required.

### Assessment of methodological quality of studies

The quality of the studies included in this review was assessed using the critical appraisal tools developed by the Joanna Briggs Institute [12]. Specifically, the checklist designed for RCTs, comprising 13 items, was utilized. Each item in the checklist was evaluated using four response options: “Yes,” “No,” “Unclear,” and “Not Applicable.” Methodological quality was initially assessed by the authors independently and then evaluated collaboratively to reach consensus.

The results are presented as “Quality Scores” in Table 1.

### Risk of bias assessment (JBI)

All seven studies met more than 50% of the 13 quality criteria, indicating an acceptable level of methodological rigor (see Table 1). The overall findings of the meta-analysis are therefore based on studies with a low-to-moderate risk of bias.

Among the included studies, Chang et al. (2016) met 12 out of 13 quality criteria, indicating very high methodological quality [13]. Chang et al. (2017) followed closely, fulfilling 11 of the 13 criteria, and was therefore evaluated as being of high quality [14]. Roberts et al. (2022) met 10 of the criteria, demonstrating moderate to high quality with some limitations [15]. Three studies—Guo & Li (2021), Rabiei et al. (2024), and Ritzi et al. (2017)—each met 9 out of 13 criteria, reflecting moderate methodological quality with notable areas for improvement [16–18]. Schottelkorb et al. (2019) also fulfilled 9 criteria; however, 4 items were not assessed due to missing information [19]. As a result, the study’s overall quality rating remains incomplete, though the available data suggest a moderate level of quality. The most common methodological limitations identified across the included studies were related to inadequate reporting of allocation concealment procedures, lack of blinding of outcome assessors, and the absence of intention-to-treat (ITT) analyses. These limitations were particularly evident in studies with moderate quality scores (i.e., those meeting 9 out of 13 criteria) and may have introduced a risk of bias, potentially affecting the reliability of the findings. Therefore, these issues should be taken into account when interpreting the pooled effect sizes in meta-analytic evaluations.

Despite these shortcomings, several strengths were noted across the studies. The consistent use of standardized outcome measures, the inclusion of comparable intervention and control groups, and the structured delivery of intervention protocols contribute positively to the internal validity of the evidence base. Moreover, studies with higher methodological quality—such as Chang et al. (2016) and Chang et al. (2017)—tended to report larger and more consistent effect sizes, particularly in domains related to social skills (Table 2). This observation supports the assertion that methodological rigor may significantly influence the strength and consistency of intervention outcomes in meta-analytic syntheses [20].

**Table 2:** Methodological Quality Assessment of Included Studies According to JBI Criteria

Study	Type of research	Q1	Q2	Q3	Q4	Q5	Q6	Q7	Q8	Q9	Q10	Q11	Q12	Q13	Quality
Chang et al., (2016)	RCT	✓	✓	✓	?	✓	✓	✓	✓	✓	✓	✓	✓	✓	12/13
Chang et al., (2017)	RCT	✓	✓	✓	?	✓	✕	✓	✓	✓	✓	✓	✓	✓	11/13
Guo & Li, (2021)	RCT	?	✓	✓	✕	✕	✕	✓	✓	✓	✓	✓	✓	✓	9/13
Rabiei et al., (2024)	RCT	✕	✓	✓	✕	✕	✕	✓	✓	✓	✓	✓	✓	✓	9/13
Ritzi et al., (2017)	RCT	✕	✓	✓	✕	✕	✕	✓	✓	✓	✓	✓	✓	✓	9/13
Roberts et al., (2022)	RCT	✓	✓	✓	✕	✕	✕	✓	✓	✓	✓	✓	✓	✓	10/13
Schottelkorb et al., (2019)	RCT	✓	✓	✓	✕	✕	✕	✓	✓	✓	-	-	-	-	10/13

✓: Yes, ✕: No, ?: Unclear, NA: Not Applicable

### Data synthesis

CMA Ver. 2 was employed for statistical computations. Heterogeneity of the studies included was analyzed using Higgins’ I^2^ statistic and the Chi-square (χ²) test. I^2^ > 50% was interpreted as indicating substantial heterogeneity.

Analyses of studies with I^2^ > 50% and *p*>0.1 were conducted employing using a random-effects model, whereas those with I^2^≤ 50% and *p*>0.1 were analyzed employing a fixed-effects model [21]. Tau-squared (Tau²) statistics were calculated to evaluate between-study variance and heterogeneity. To determine the effect size across studies using different measurement instruments but targeting the same outcome, standardized mean difference (SMD) with a 95% confidence interval (CI) was calculated. Forest plots were prepared to visually represent the SMD values along with their 95% CIs. The overall effect size was obtained by computing the mean of the D values, which were then converted into Z-scores to assess statistical significance. Publication bias was tested employing Egger’s test and was visually represented with a funnel plot. All values were two-sided, and statistical significance at *p* < 0.05 [20, 22]. Additionally, Mazumdar’s rank correlation test and Begg’s test were performed to determine potential publication bias.

To further explore variability across intervention approaches, subgroup and moderator analyses were conducted according to intervention type and practitioner-related characteristics when sufficient data were available. These analyses were performed to examine whether conceptual and procedural differences among play-based intervention models contributed to variations in treatment outcomes. In addition, because the included studies used different instruments to assess social and play skills (e.g., standardized scales, observational assessments, and report-based measures), standardized mean differences were preferred to ensure comparability of effect sizes across studies.

## Results

All studies included in this analysis are RCTs. The total sample size across the studies is 411 participants (intervention: 209; control: 202) (Table 1). This indicates that the findings are derived from studies with an overall acceptable level of methodological quality, although variability in rigor across studies was observed.

All studies included in this meta-analysis met > 50% of the criteria on the quality of evidence assessment tool (Table 1). This is significant in demonstrating that the findings reported in this study are based on research with an acceptable level of methodological quality.

The publication bias in this meta-analysis was tested employing a Funnel Plot and Egger’s Regression Test.

### Meta-analysis results on the effect of play therapy on social skill levels

**Fig. 2:**
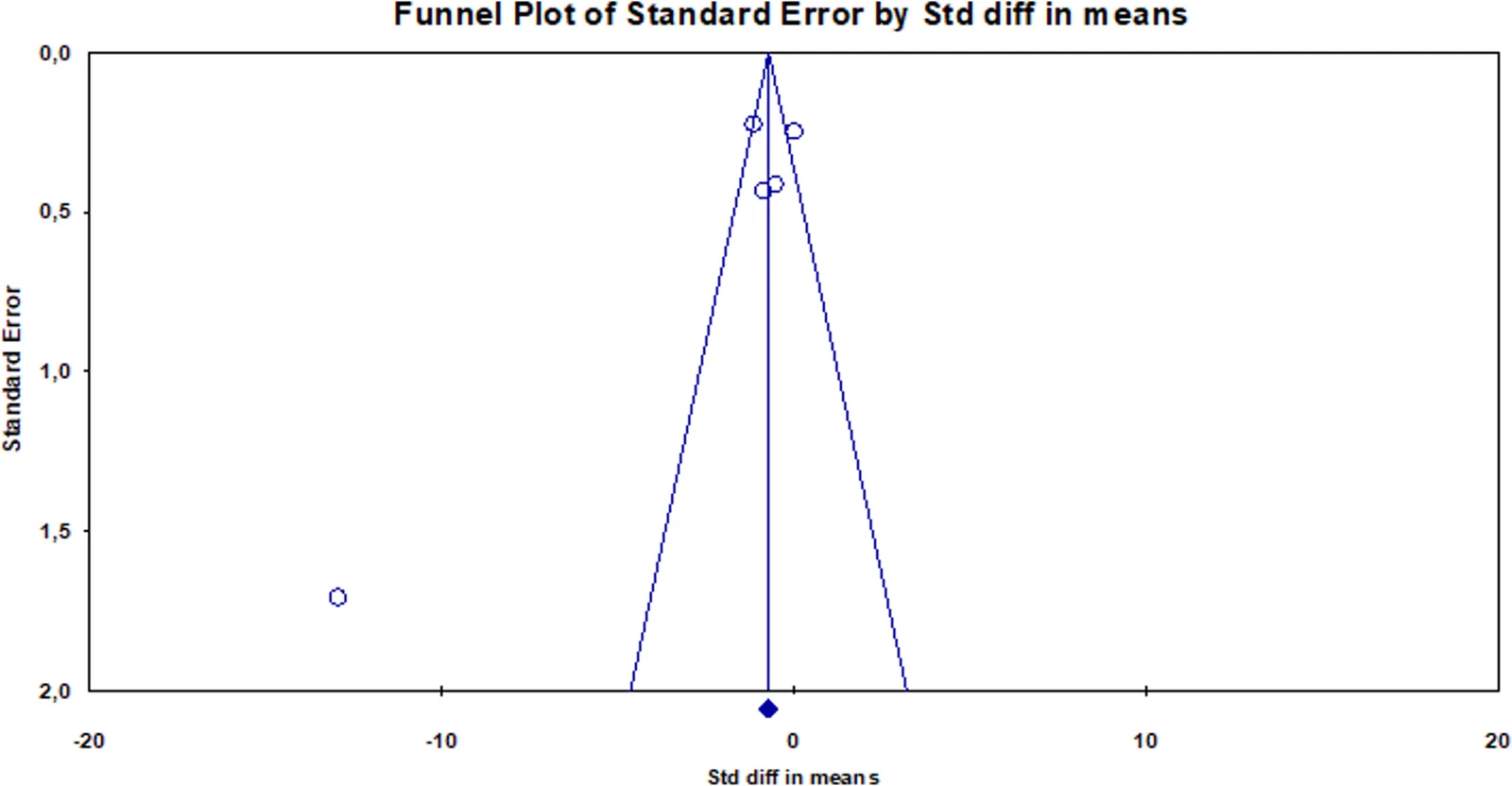
Funnel Plot of the Studies

Publication bias in this dataset was analyzed by employing Egger’s method, yielding no evidence of bias. Given the results of Egger’s regression, the intercept (B₀) was − 5.89845, with a 95% confidence interval of [−15.85380, 4.05691], t = 1.88557, df = 3, and a two-tailed *p* = 0.15584, indicating no significant evidence of publication bias (*p* = 0.15584). Moreover, the funnel plot in Fig. 2 confirms the absence of publication bias.

**Fig. 3:**
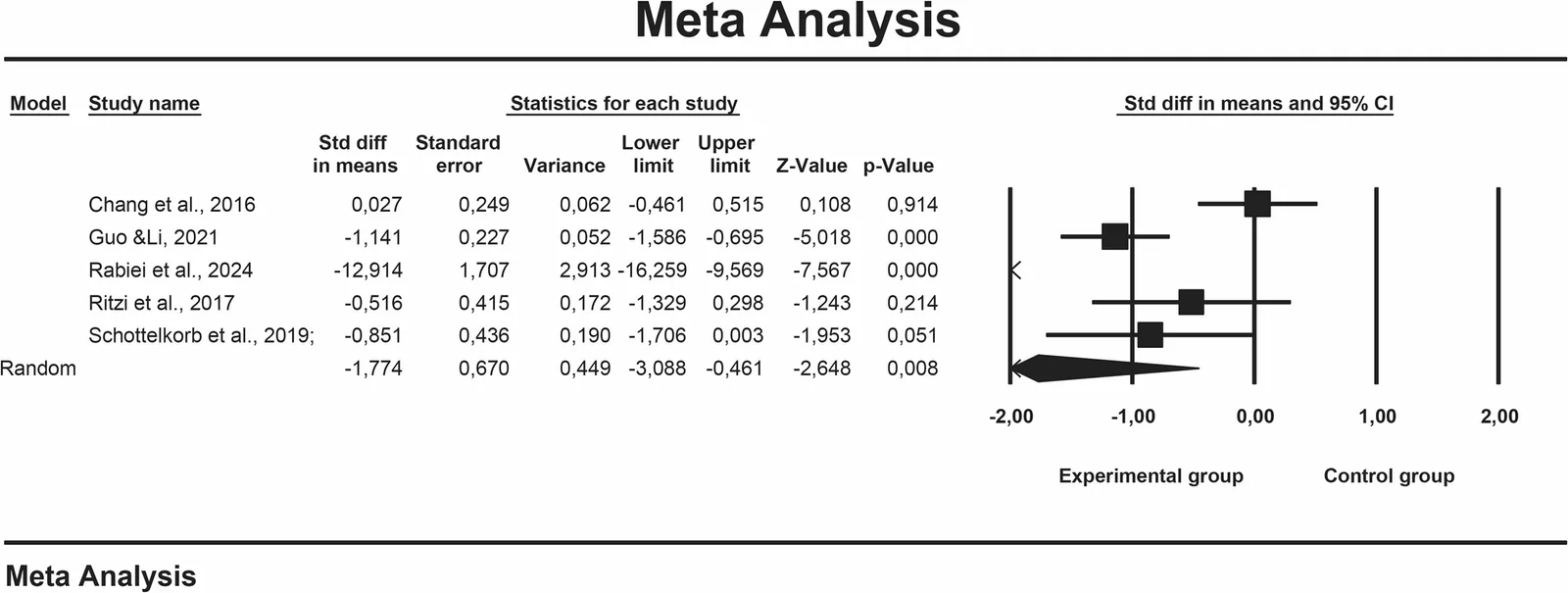
Forest Plot of the Studies

Five RCTs (233 participants in total) evaluated social skill scores after intervention by using the SRS-2, GARS-2, ABC, ESCS, and the Child Behavior Checklist - Parent Report (Chang et al., 2016; Guo & Li, 2021; Rabiei et al., 2024; Ritzi et al., 2017; Schottelkorb et al., 2019). Because of considerable heterogeneity, the random-effects model was applied in the meta-analysis (I^2^ = 93.730%, Q = 63.799; *p* < 0.05). The findings suggest that play-based interventions are associated with improvements in social skills in children with ASD (SMD=−1.774, 95% CI: −3.088 to −0.461; Z=−2.648, *p* = 0.008) (see Fig. 3).

### Meta-analysis results on the effect of play therapy on play skill levels

**Fig. 4:**
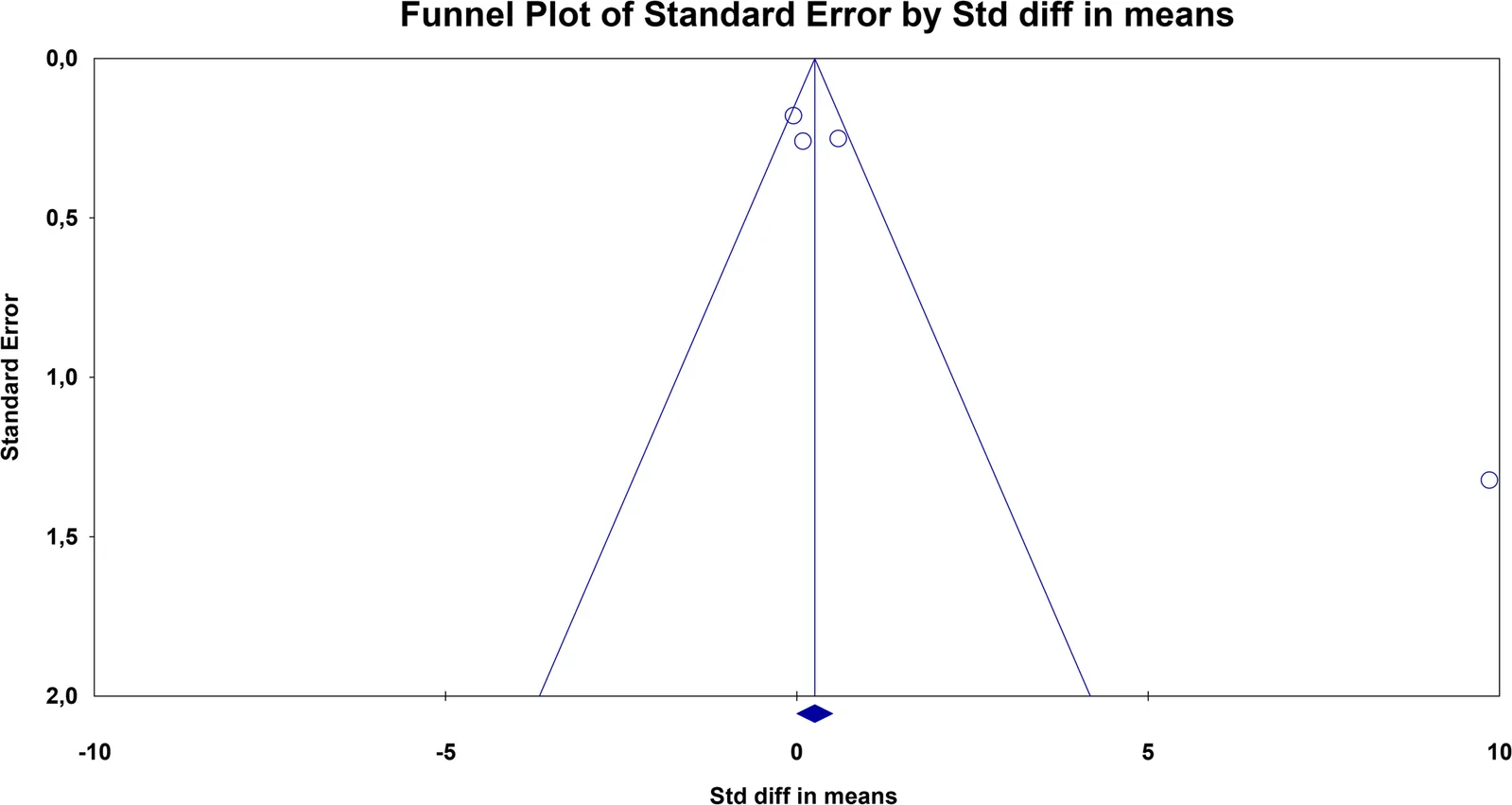
Funnel Plot of the Studies

Publication bias in this dataset was assessed using Egger’s test, yielding no evidence of publication bias. Given the results of Egger’s method, the intercept (B₀) was 8.30928 with a 95% CI ranging between 1.93197 and 14.74659; t = 5.60001, df = 2, and the two-tailed *p* = 0.05044, indicating no significant publication bias (*p* = 0.05044). Furthermore, this finding was visually corroborated by the funnel plot (see Fig. 4).

**Fig. 5:**
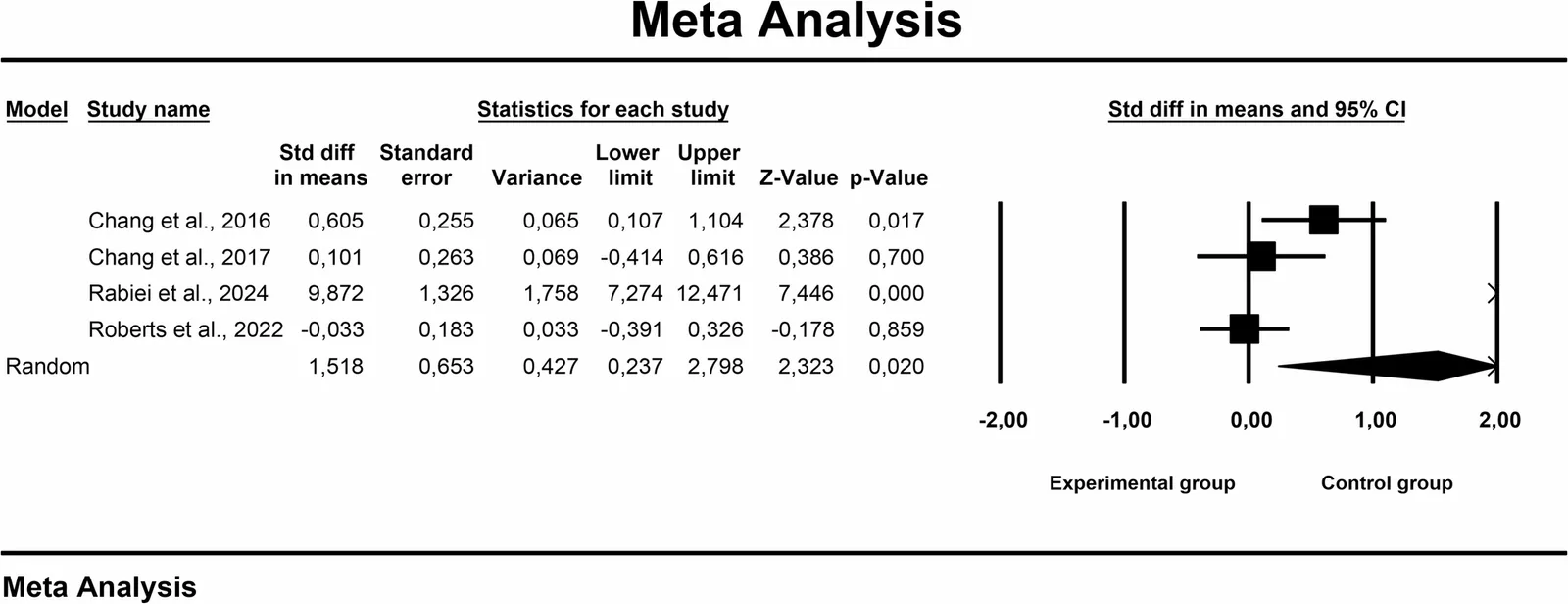
Forest Plot of the Studies

Four RCTs involving a total of 274 participants evaluated post-intervention play skill scores using the JASPER framework, the SPA, and the Lincoln-Oseretsky Motor Development Scale (Chang et al., 2016; Chang et al., 2017; Rabiei et al., 2024; Roberts et al., 2022). Based on the findings of these studies, a meta-analysis was conducted by utilizing the random-effects model because of substantial heterogeneity (I²=94.768%; Q = 57.335; *p* < 0.05). The results indicate that play-based interventions are associated with a significant increase in play skill levels among children with ASD (SMD: 1.518, 95% CI: 0.237 to 2.798; Z = 2.323, *p* = 0.020) (see Fig. 5).

### GRADE assessment

Two authors (Y.Ç.B. and E.D.E.) assessed the quality of the included studies independently by using the JBI checklist and performed a GRADE evaluation for the outcomes analyzed in the meta-analysis. Any discrepancies between the reviewers were resolved via discussion, and if needed, consultation with a 3rd reviewer.

For the outcome of social skills, the evidence was rated as moderate quality. While all five studies were RCTs, the high level of heterogeneity (I²=93.7%) led to a downgrade in quality. Although the pooled effect size was large, the observed heterogeneity suggests that the magnitude of effect may vary substantially across intervention types and study contexts, resulting in a final classification of moderate quality.

For the outcome of play skills, the evidence was rated as low quality. Although 4 of the studies included were RCTs, the evidence was downgraded due to very high heterogeneity (I²=94.8%) and wide confidence intervals (95% CI: 0.237 to 2.798), which suggest imprecision in the results (Table 3). Therefore, the pooled effect size should be interpreted cautiously. Additional high-quality trials are necessitated to confirm the effect of play-based interventions on play skill development in children with ASD.

**Table 3:** GRADE Summary of Findings

Outcome	№ of Participants (studies)	Effect Size (95% CI)	Certainty of the Evidence (GRADE)	Comments
Social skills	233 participants (5 RCTs)	SMD: −1.774 (−3.088 to −0.461)	⬤⬤⬤◯ Moderate	Downgraded for heterogeneity; upgraded for large effect and RCT design.
Play skills	274 participants (4 RCTs)	SMD: 1.518 (0.237 to 2.798)	⬤⬤◯◯ Low	Downgraded for heterogeneity and imprecision; small number of studies.

### Moderator analyses results

Moderator analyses were conducted to assess the effects of play-based interventions on social and play skills among children with ASD. These analyses included subgroup analyses and meta-regression techniques and were performed to explore potential sources of heterogeneity across studies [23]. In the present study, moderators were examined under three main categories: duration of intervention, type of intervention, and practitioner.

Moderator analyses regarding the intervention duration were conducted over 12, 15, 20, 24, and 48 sessions. The overall effect size was calculated to be −0.222 (SE = 0.130, *p* = 0.088), which was not significant. However, interventions lasting 15 sessions were associated with a statistically significant effect, highlighting the potential influence of intervention duration on outcomes.

In the moderator analysis regarding the intervention type, the average effect size was determined to be −0.362 (SE = 0.105) (*p* = 0.006), indicating that intervention type significantly moderated effect sizes. Among the interventions, CCPT, ISPT, and SIBPT were associated with larger effect sizes compared to other play-based approaches.

In the moderator analysis regarding the type of practitioner, the effect size for interventions delivered by trained therapists was calculated to be −1.141 (SE = 0.227, *p* < 0.001). The overall average effect size across different practitioner types was − 0.315 (SE = 0.114, *p* = 0.006) (Table 3). These findings suggest that practitioner expertise may play a critical role in the effectiveness of play-based interventions (Table 4).

**Table 4:** Moderator analysis

Moderator	The number of studies	Effect size	Standard error	Lower limit	Upper limit	*p*
Intervention period						
12 sessions	2	−0.5610	0.584	−1.706	0.583	**0.336**
15 sessions	1	−12.914	1.707	−16.259	−9.569	**0.000**
20 sessions	1	−0.516	0.415	−1.329	0.298	0.214
24 sessions	1	−0.851	0.436	−1.706	0.003	0.051
48 sessions	2	0.011	0.150	−0.283	0.305	0.941
Total	7	−0.222	0.130	−0.478	0.033	0.088
Type of Intervention						
Caregiver-Mediated Social Communication Support	1	−0.033	0.183	−0.391	0.326	0.859
CCPT	2	−0.675	0.301	−1.264	−0.086	**0.025**
ISPT	1	−1.141	0.227	−1.586	−0.695	**0.000**
JASPER	2	0.062	0.181	−0.292	0.416	0.731
SIBPT	1	−12.914	1.707	−16.259	−9.569	**0.000**
Total	7	−0.362	0.105	−0.567	−0.157	**0.001**
Implementer						
Caregivers/educators	1	−0.033	0.183	−0.391	0.326	0.859
Play therapists	2	−0.124	0.297	−0.706	−0.416	0.677
Researchers/therapists	2	−6.770	6.030	−18.588	5.049	0.262
Teachers	1	0.027	0.249	−0.461	0.515	0.914
Therapy specialists	1	−1.141	0.227	−1.586	−0.695	**0.000**
Total	7	−0.315	0.114	−0.539	−0.092	**0.006**

Collectively, these results suggest that heterogeneity in intervention characteristics and practitioner expertise contributes to variability in outcomes, emphasizing the importance of considering these moderators when interpreting the pooled results of play-based interventions for children with ASD.

## Discussion

This meta-analysis aims to evaluate the effect of play-based interventions on the social and play skills of children with ASD. The findings indicate that play-based interventions are associated with significant improvements in both social skills (*p* = 0.008) and play skills (*p* = 0.020). The notably high standardized mean difference (SMD = 1.518) suggests a potentially meaningful therapeutic effect, although the substantial heterogeneity across studies indicates that effect magnitude may vary depending on intervention characteristics and implementation context.

Subgroup analyses showed that factors such as the intervention type and the qualifications of the practitioner play an important role in moderating the effectiveness of treatment. Specifically, interventions such as CCPT, ISPT, and SIBPT were associated with comparatively larger effect sizes. Furthermore, interventions implemented by qualified therapists were associated with enhanced therapeutic outcomes, suggesting that practitioner expertise may contribute significantly to intervention success.

These results are consistent with the existing literature. For example, in their meta-analysis, Tiede and Walton (2019) reported that naturalistic developmental behavioral interventions produced significant effects particularly in the areas of social interaction (g = 0.65) and cognitive development (g = 0.48) (Tiede & Walton, 2019). This finding supports the potential benefits of structured play-based developmental interventions in early childhood development [24]. Similarly, in their systematic review and meta-analysis, Kent et al. (2020) determined that play-based interventions targeting children with ASD aged 2–12 produced a small but significant effect size (Hedges’ g = 0.439) [8]. In a systematic review carried out by Dijkstra-de Neijs et al. (2021), the majority of the 32 RCTs examined reported improvements in social interaction, communication, daily functioning, and play behaviors [25]. However, the high risk of bias identified in many of these studies limits the generalizability of their findings. This limitation provides an important framework for interpreting the variability in effect sizes observed in the present meta-analysis, particularly across different intervention types and practitioner characteristics.

In addition, in a systematic review, Schaaf et al. (2019) identified Ayres Sensory Integration (ASI) therapies as evidence-based practices for children with ASD aged 4–12 years [26]. This finding supports the higher effect sizes observed in the present study for interventions incorporating ASI components. Therefore, the integration of sensory-based interventions into therapeutic processes may represent a promising strategy for supporting the development of children with ASD.

From a clinical perspective, improvements in social and play skills are particularly relevant because these skills are closely associated with peer interaction, adaptive functioning, and participation in naturalistic social environments. Although most included studies measured outcomes in structured therapeutic or assessment settings, enhancements in these foundational developmental skills may facilitate broader functional and social participation. Nevertheless, further longitudinal studies are needed to evaluate the extent to which these improvements generalize to real-world settings and are sustained over time.

Taken together, these findings suggest that structured and child-centered play therapies represent potentially beneficial and feasible intervention approaches for fostering the social-emotional development of children with ASD. The present findings also underscore the role of methodological quality in shaping the strength of evidence. Studies with moderate-to-high quality scores contributed more robustly to the overall effect sizes, particularly in relation to social skill outcomes. In contrast, findings derived from lower-quality studies were characterized by wider variability, aligning with previous concerns regarding the interpretation of heterogeneous results in meta-analytic synthesis [20]. Therefore, the pooled results should be interpreted cautiously, considering the variability in intervention models, outcome measures, and study designs. Additionally, the relatively small number of studies included in this meta-analysis may limit the robustness and generalizability of the findings. In particular, the limited number of eligible RCTs may reduce the stability of subgroup and moderator analyses and increase sensitivity to study-level variations. Therefore, the findings should be interpreted with caution until supported by larger and methodologically rigorous studies.

## Conclusion and suggestions

Findings obtained in this study suggest that play-based interventions may represent beneficial therapeutic approaches for enhancing the social and play skills of children with ASD. In particular, structured and child-centered approaches (e.g., CCPT, ISPT) were associated with comparatively stronger outcomes across included studies. Developing individualized play-based intervention programs tailored to children’s needs and ensuring that the intervention process is conducted by trained professionals may positively influence therapeutic outcomes. Incorporating play-based interventions into early intervention programs may contribute to supporting the social participation and adaptive skills of children with ASD.

Future studies should focus on monitoring the long-term effects of play-based interventions in order to evaluate the sustainability and generalizability of post-therapy gains. Moreover, increasing the number of high-quality RCTs comparing different types of play-based therapeutic approaches could help identify which intervention models are most effective for specific child profiles. Studies examining how these interventions can be integrated with parental involvement, environmental factors, and educational support would also significantly contribute to the field. Therefore, it is recommended that future studies include more diverse and larger samples, with attention to cultural context and intervention standardization.

Considering the substantial heterogeneity across studies and variability in intervention characteristics, the pooled findings should be interpreted cautiously. Further rigorously designed trials are needed to strengthen the evidence base and clarify the comparative effectiveness of different play-based intervention models.

## Limitations

This meta-analysis has several limitations. First, variations in the methodological quality and sample sizes of the included studies may limit the generalizability of the observed effect sizes. Second, there was considerable heterogeneity in the duration, frequency, and content of the play-based interventions, which may have influenced the interaction effects in the analyses. Third, the included studies encompassed different theoretical frameworks and therapeutic models, ranging from child-centered play therapy approaches to structured developmental-behavioral interventions utilizing play as a therapeutic medium. Although moderator analyses were conducted to explore potential sources of variability, this diversity may limit the interpretation of a single pooled effect size. Fourth, outcome measures assessing social and play skills varied across studies, and this variability may have influenced the comparability of effect sizes despite the use of standardized mean difference calculations. Although analyses were performed to account for publication bias, the exclusion of unpublished studies may have restricted the comprehensiveness of the findings. Additionally, the relatively small number of eligible RCTs included in the meta-analysis may reduce the statistical power and stability of subgroup and moderator analyses. Finally, most included studies evaluated outcomes in structured therapeutic or assessment settings, which may limit conclusions regarding the long-term sustainability and real-world generalizability of intervention effects.

## Data Availability

The data that support the findings of this study are available from the corresponding author upon reasonable request.
